# Supplementary material for: Clinical and Experimental Determination of Protection Afforded by BCG Vaccination against Infection with Non-Tuberculous Mycobacteria: A Role in Cystic Fibrosis?
Source: Vaccines (Basel). 2023 Aug 1;11(8):1313. doi: 10.3390/vaccines11081313 (PMC10459431; doi:10.3390/vaccines11081313)
Supplement: Supplementary file 1 [file vaccines-11-01313-s001.zip › vaccines-2510426-supplementary.pdf]

Supporting Information

Clinical and experimental determination of protection afforded by BCG vaccination against infection with non-tuberculous mycobacteria: a role in Cystic Fibrosis?

Warner *et. al*.

Supplementary Table S1. Monoclonal antibodies used for $CD4^+$ T cell analysis.

Marker	Clone	Fluorochrome	Dilution	Manufacturer
B220	PA3-6B2	PerCP/Cy5.5	1 in 200	BD Pharmingen
CD4	RM4-5	AF700	1 in 200	BD Pharmingen
CD44	IM7	PE/Cy7	1 in 300	BD Pharmingen
CD62L	MEL-14	PE	1 in 300	BioLegend
CD8a	53-6.7	APC-Cy7	1 in 200	BD Pharmingen
FcRIII/II	2.4G2	NA	1 in 200	BD Pharmingen
KLRG-1	2F1/KLRG1	FITC	1 in 300	BioLegend
RorγT	Q31-378	PECF594	1 in 200	BD Horizon
Tbet	4B10	APC	1 in 200	BioLegend

Supplementary Table S2. Monoclonal antibodies used to assess antigen-specific cytokine production.

Marker	Clone	Fluorochrome	Dilution	Manufacturer
CD4	RM4-5	AF700	1 in 200	BD Pharmingen
CD44	IM7	FITC	1 in 300	BD Pharmingen
CD8a	53-6.7	APC-Cy7	1 in 200	BD Pharmingen
FcRIII/II	2.4G2	NA	1 in 200	BD Pharmingen
IFNγ	XMG1.2	PE/Cy7	1 in 300	BD Pharmingen
IL-17	TC11-18H10	Pacific Blue	1 in 200	BD Horizon
IL-2	JES6-5H4	PE	1 in 200	Miltenyi Biotec
TNF	MP6-XT22	PerCP/Cy5.5	1 in 200	BD Pharmingen

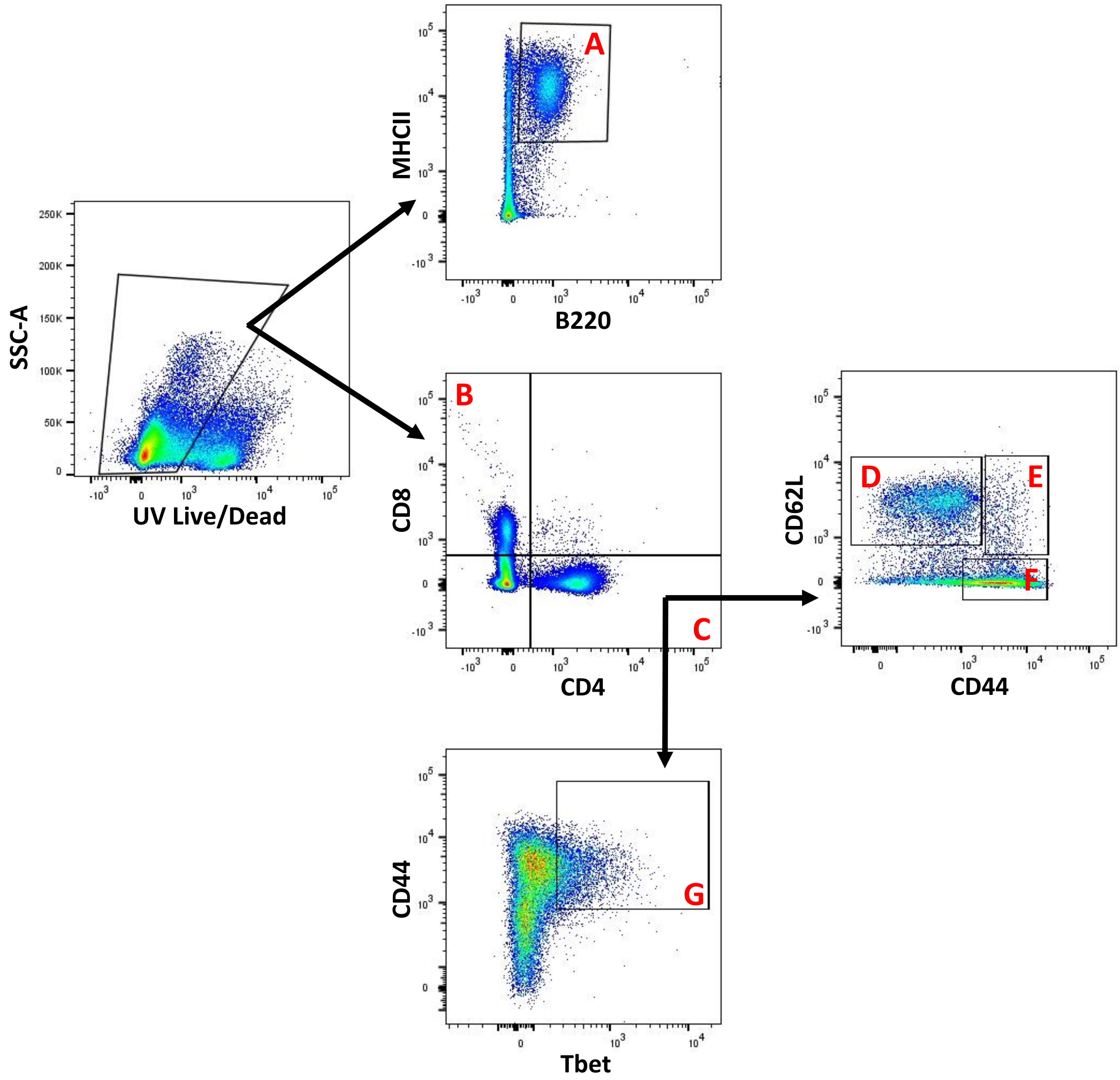

Supplementary Figure S1. Gating strategy for expression of cytokines and transcription factors by $CD4^+$ T cells
